# The effect of vitamin D supplementation on markers of insulin resistance in women with polycystic ovarian syndrome: a systematic review

**DOI:** 10.1007/s00394-024-03489-6

**Published:** 2024-09-14

**Authors:** Georgia Kohlhoff, Richard Kirwan, Sohail Mushtaq

**Affiliations:** 1 Faculty of Medicine, Dentistry and Life Sciences, University of Chester, Chester, UK; 2 Research Institute for Sport and Exercise Sciences, Liverpool John Moores University, Liverpool, UK

**Keywords:** Polycystic ovary syndrome, PCOS, Insulin resistance, Vitamin D, 25(OH)D, Glycemic control, Diabetes

## Abstract

**Background:**

Insulin resistance (IR) is a common pathology in women with polycystic ovarian syndrome (PCOS) involved in increased rates of cardiometabolic disease such as diabetes and cardiovascular disease. Low serum vitamin D is often associated with insulin resistance but there is no consensus on whether vitamin D supplementation can ameliorate markers of IR in PCOS.

**Objectives:**

We assessed evidence on the effects of vitamin D supplementation (≥ 1000 IU/day), without the use of additional supplements or other pharmacological treatments known to affect IR, on markers of IR and glycemic control in women with PCOS.

**Design:**

A systematic search was conducted using PubMed, Medline and Web of Science databases from January 2000 up to November 2023. Randomized controlled trials that assessed the effects of vitamin D supplementation in women with PCOS, on fasting glucose, fasting insulin, glycated haemoglobin (HbA1c) or homeostatic model assessment for insulin resistance (HOMA-IR) were included.

**Results:**

9 studies were identified. Study populations ranged from 28 to 180 participants, with mean ages ranging from 22 to 30 years. Daily vitamin D doses ranged from 1714-12,000 IU. Of the included studies, 3 reported statistically significant reductions in fasting glucose, 2 reported reductions in fasting insulin, 2 reported reductions in HOMA-IR, none reported reductions in HbA1c and 5 reported no differences in any of the relevant outcomes.

**Conclusions:**

In conclusion, in RCTs of vitamin D supplementation in women with PCOS, the majority of studies do not report statistically significant improvements in fasting glucose, fasting insulin, HbA1c or HOMA-IR. However, as a minority of studies report some statistically significant results, further investigation may be warranted.

**Registry:**

PROSPERO ID: CRD42023486144

**Supplementary Information:**

The online version contains supplementary material available at 10.1007/s00394-024-03489-6.

## Introduction

Polycystic ovary syndrome (PCOS) is the most common endocrine disorder affecting women of reproductive age, affecting approximately one fifth of women of reproductive age [1] and it is the leading cause of anovulatory infertility in women [2]. PCOS is characterized by a cluster of pathologies, including irregular menstrual cycle, hyperandrogenism, and polycystic ovaries [1]. Insulin resistance (IR), a pathological state in which the body's tissues become resistant to the effects of insulin, leading to hyperinsulinemia and compensatory hyperglycemia, is a common feature of PCOS, with up to 70% of women with PCOS exhibiting some degree of IR (Dunaif, 1997). This IR is believed to contribute to the elevated risk of, obesity, diabetes and cardiovascular disease in women with PCOS [3, 4].

Vitamin D has been implicated in the pathogenesis of insulin resistance and type 2 diabetes mellitus (T2DM), due to its effects on insulin secretion and sensitivity, inflammation, and calcium homeostasis [5]. In European populations, vitamin D insufficiency (serum 25-hydroxy vitamin D [25(OH)D] concentration <50 nmol/L) is believed to affect up to 40% of individuals [6] and vitamin D deficiency (25(OH)D concentration <30 nmol/L) is considered to be a global health concern [7, 8]. Vitamin D insufficiency is also common among women with PCOS, with some studies reporting a prevalence of up to 70% [9].

Given the potential interplay between vitamin D and IR in the context of PCOS, several randomized controlled trials (RCTs) have investigated the effect of vitamin D supplementation on markers of IR. However, while some studies have demonstrated a positive effect of supplementation [10, 11], others have shown no such benefit [12, 13]. Therefore, to investigate the role of vitamin D supplementation in ameliorating markers of insulin resistance we completed a systematic review of RCTs assessing the effect of vitamin D, without the use of additional supplements or other pharmacological treatments known to affect IR, on fasting glucose, fasting insulin, glycated haemoglobin (HbA1c) or homeostatic model assessment for insulin resistance (HOMA-IR) in women with PCOS.

## Methods

The systematic review protocol was performed in accordance with the Preferred Reporting Items for Systematic Reviews and Meta-Analyses (PRISMA) statement guidelines [14] and following the criteria outlined in the Cochrane Handbook for Systematic Reviews of Interventions [15]. The protocol was registered with PROSPERO (ID: CRD42023486144).

### Search strategy

PubMed, Medline (EBSCO) and Web of Science databases were searched from January 2000 until November 30th, 2023, limiting searches to human RCTs in English language. The PICO (Population/ intervention/ comparison/ outcome) to identify relevant papers was as follows: P (adults ≥18 years), I (vitamin D, ≥ 1000 IU/day), C (placebo), and O (fasting glucose, fasting insulin, HbA1c or HOMA-IR). The following search strategy and keywords were used, as presented, in each database: ((vitamin D) OR (25OHD) OR (25(OH)D) OR (*calciferol)) AND ((insulin resistance) OR (insulin sensitivity) OR (glucose control) OR (glycemic control) OR (hba1c) OR (homa-ir) OR (insulin) OR (glucose)) AND ((polycystic ovary syndrome) OR (polycystic ovarian syndrome) OR (pcos)).

### Study selection criteria

Two independent investigators (GK and RK) screened titles and abstracts for relevant studies. Only RCTs that assessed the effects of vitamin D supplementation in adult women (mean age ≥ 18 years) with PCOS, on common measures of glycemic control/IR were included. Acceptable measures of glycemic control/IR were limited to fasting glucose, fasting insulin, HbA1c or HOMA-IR due to their frequency of use in the literature as measures that can be determined with single blood tests [16–18]. Studies were required to specify duration and only those with an intervention of a minimum of 8 weeks duration were included as previously published literature has indicated that such durations of supplementation with 1000–2000 IU/day of vitamin D3 may be required to achieve sufficient levels plasma levels, from a deficient state [19]. Interventions with dietary modification, supplementation of additional vitamins/minerals or pharmacological treatments known to affect IR were excluded. Studies in populations suffering from pathologies other than sarcopenia and frailty (*e.g.,* cancer, cardiovascular disease, diabetes etc.) were also excluded. Study inclusion and exclusion criteria are summarized in Table 1.

**Table 1:** Inclusion and exclusion criteria

Inclusion Criteria	Exclusion Criteria
Population	Population
Age >18 years	Individuals with comorbidities including cardiovascular disease, type 2 diabetes, cancer, Non-Alcoholic Fatty Liver Disease, chronic kidney disease etc.
PCOS	
Intervention	Intervention
Randomized controlled trial	Other vitamin/mineral supplements
Supplementary vitamin D >1000IU/day	Pharmacological treatments
Non-supplemented control or placebo	
Minimum duration of 6 weeks	
Primary outcomes	
HbA1c	
HOMA-IR	
Fasting serum glucose	
Fasting serum insulin	
Other	Other
Full paper	Protocol papers
English language	Abstract only

### Data extraction

Two investigators (GK and RK) independently extracted data from the original publications. Data on age, country of intervention, baseline and endpoint serum vitamin D level (where available), vitamin D dosage and frequency, and intervention duration, and primary outcomes were extracted. In order to avoid double counting of control arms, where multiple treatment arms were used with only one control group, priority was given to treatment arms with higher dosages of vitamin D. Discrepancies were resolved by group consultation (GK, RK, and SM) until consensus was reached.

### Risk of bias assessment

Risk of bias of RCTs was evaluated independently by two investigators (GK and RPK). The assessment was performed at the study level with the revised Cochrane risk of bias tool (RoB 2) which grades the risk of selection, performance, attrition, detection, and reporting biases [20]. This tool assesses whether a study has a low, unclear, or high risk of bias. Differences in opinion were resolved by group consultation (GK, RK, and SM) until consensus was reached.

## Results

### Flow and characteristics of included studies

**Fig. 1:**
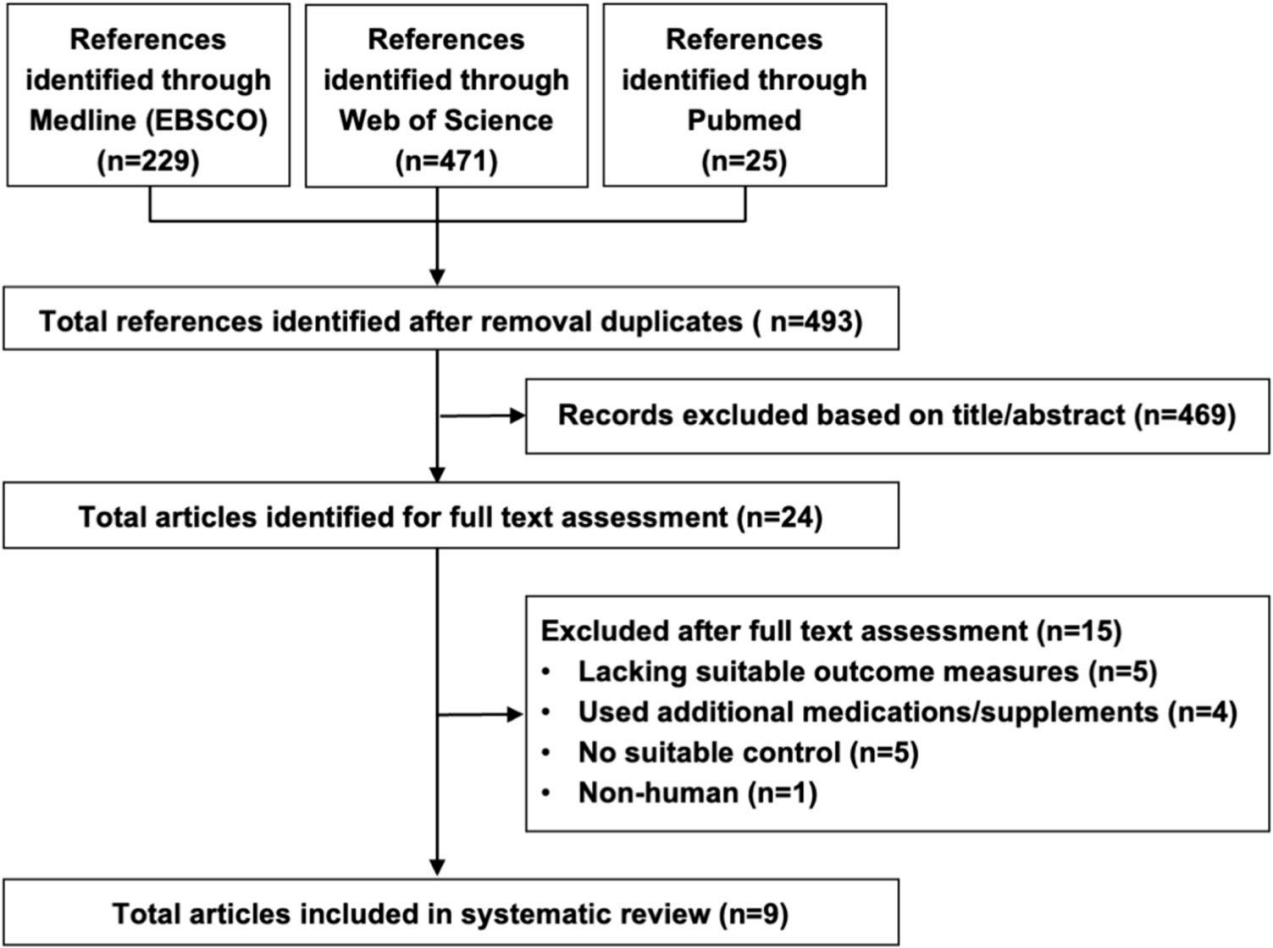
PRISMA flow diagram of study selection through the systematic review process. PRISMA, Preferred Reporting Items for Systematic Reviews and Meta-Analysis

Figure 1 shows the flowchart of studies in the review process. After removal of duplicates, 493 records were identified by the initial literature search. Through review of titles and abstracts, 24 potentially relevant articles were selected for full-text evaluation. Subsequently, 9 eligible randomized controlled studies met the inclusion criteria [10–13, 21–25].

The characteristics of the studies included in the systematic review are presented in Table 2. Briefly, studies ranged in size from 28 to 180 participants per study, with mean ages of participants ranging from 22 to 30 years. Location of interventions ranged from Iran (4 studies) [10, 11, 21, 25], USA (2 studies) [12, 23], Austria (1 study) [13], India (1 study) [22], and UK (1 study) [24]. Study durations ranged from 8 weeks (4 studies) [10, 11, 21, 23] to 12 weeks (4 studies) [12, 22, 24, 25], and 24 weeks (1 study) [13]. In terms of body mass index (BMI), participants ranged from normal BMI (18.5-24.9 kg/m^2^) (1 study) [25], overweight (25.29.9 kg/m^2^) (5 studies) [11, 13, 21–23], and obese (>30 kg/m^2^) (2 studies) [12, 24]. Abootorabi et al [10] did not report data on BMI. Based on cut-off values for insulin resistance (defined as HOMA-IR ≥2.1) [26] all but one [23] of the included studies had a insulin resistant intervention or control group at baseline.

**Table 2:** Participant characteristics and intervention details of the 9 included studies

Author (Year)	Intervention group	n	Mean age (years)	Country	BMI (kg/m^2^)	Vitamin D dose (IU) /Control	Frequency of vitamin D treatment	Vitamin D daily equivalent (IU)	Duration (weeks)	Change in serum 25(OH)D nmol/L	Main findings Intervention *v*s Control
Abootorabi et al. (2018) [10]	Intervention	22	26.2	Iran	–	50,000 (Vitamin D_3_)	1/week	7143	8	21.6 ± 10.8 to 92.3 ± 20.9	FPG ↓ No difference in FSI or HOMA-IR
	Control	22	22.8		–	Placebo				24.5 ± 12.8 to 33.4 ± 17.8	
Ardabili et al. (2012) [21]	Intervention	30	26.8	Iran	29.10 ± 4.62	50,000 (Vitamin D_3_)	1/20 days	2500	8	17.3 ± 7.0 to 58.5 ± 15.3	No difference in FPG, FSI or HOMA-IR
	Control	30	27		28.28 ± 3.51	Placebo				18.5 ± 7.0 to 20.5 ± 5.8	
Dastorani et al. (2018) [11]	Intervention	20	29.9	Iran	27.7 ± 3.9	50,000 (Vitamin D_3_)	1/ 2 weeks	3571	8	26.3 ± 26.3 to 54.3 ± 14.8	FSI and HOMA-IR ↓ No change in FPG
	Control	20	30.1		28.4 ± 2.6	Placebo				27.5 ± 6.0 to 27.3 ± 5.3	
Gupta et al. (2017) [22]	Intervention	25	26.0	India	24.93 ± 2.81	12,000 (Vitamin D_3_)	1/ week	1714	12	46.4 ± 24.2 to 112.3 ± 22.6	FSG, FSI and HOMA-IR ↓
	Control	25	26.6		25.55 ± 1.98	Placebo				N/A	
Irani et al. (2015) [23]	Intervention	35	30.5	USA	30 ± 1	50,000 (Vitamin D_3_)	1/ week	7143	8	40.8 ± 2.3 to 108.0 ± 6.0	No difference in HOMA-IR
	Control	18	29.6		28 ± 1.6	Placebo				42.5 ± 4.5 to 43.5 ± 4.8	
Javed et al. (2019) [24]	Intervention	18	28.6	UK	35.4 ± 10.6	3,200 (Vitamin D)	1/ day	3200	12	25.6±11.4 to 90.4 ± 19.5	No difference in FPG, FPI or HOMA-IR
	Control	19	29.1		33.8 ± 7.2	Placebo				30.9 ± 11.1 to 47.6 ± 20.5	
Maktabi et al. (2017) [25]	Intervention	35	22.0	Iran	22.7 ± 3.4	50,000 (Vitamin D_3_)	1/ 2 weeks	3571	12	32.0 ± 11.3 to 68.8 ± 24.5	FPG, FPI and HOMA-IR ↓
	Control	35	23.1		24.1 ± 3.8	Placebo				36.3 ± 12.8 to 36.0 ± 13.0	
Raja-Khan et al. (2014) [12]	Intervention	13	28.2	USA	37.20 ± 4.53	12,000 (Vitamin D_3_)	1/ day	12,000	12	48.9 ± 23.7 to 168.4 ± 71.6	No difference in FPG, FPI or HOMA-IR
	Control	15	28.7		35.09 ± 9.81	Placebo				56.1 ± 17.6 to 55.5 ± 17.2	
Trummer et al. (2019) [13]	Intervention	119	25.4	Austria	27.3 ± 7.4	20,000 (Vitamin D_3_)	1/ week	2857	24	48.8 ± 16.8 to 90.2 ± 20.1	No difference in FPG, HbA1c or HOMA-IR
	Control	61	27.2		28.3 ± 7.8	Placebo				48.8 ± 17.5 to 56.8 ± 29.5	

*BMI* body mass index; *FPG* fasting plasma glucose; *FPI* fasting plasma insulin; *FSI* fasting serum insulin; *HbA1c* glycated haemoglobin; *HOMA-IR* homeostatic model assessment for insulin resistance; *IU* international units

### Vitamin D interventions

Individual doses of vitamin D ranged from 3,200 IU (1 study) [24], to 12,000 IU (2 studies) [12, 22], to 20,000 IU (1 study) [13], and to 50,000 IU (5 studies) [10, 11, 21, 23, 25].

Frequency of vitamin D dosage ranged from once per day (2 studies) [12, 24], to once per week (4 studies) [10, 13, 22, 23], to once every 2 weeks (2 studies) [11, 25], to once every 20 days (1 study) [21].

Daily vitamin D dose varied with ranges of 1000-4,999 IU per day (6 studies) [11, 13, 21, 22, 24, 25], 5000-9,999 IU (2 studies) [10, 23], and 10,000-12,000 IU (1 study) [12].

### Risk of bias assessment

**Fig. 2:**
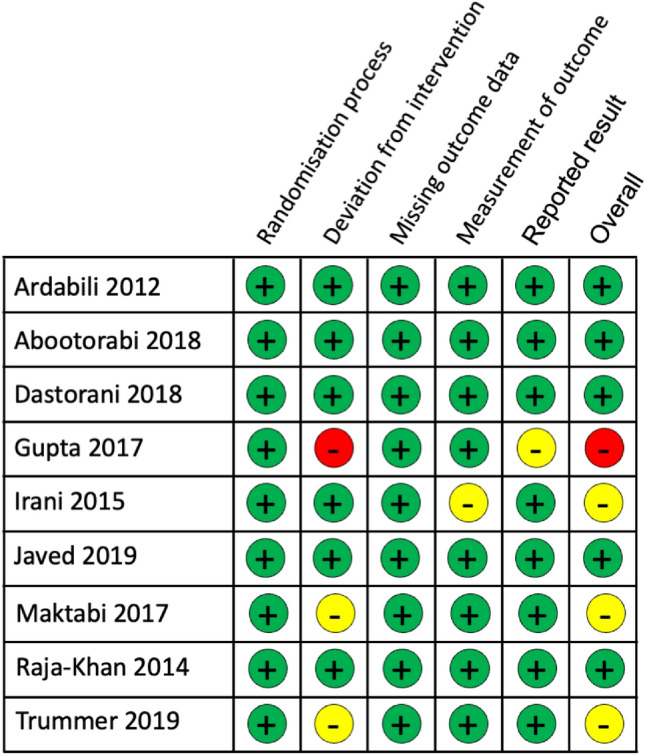
Risk of bias summary for the included studies

Risk of bias of RCTs was evaluated with the revised Cochrane risk of bias tool. This tool determined 5 studies had low risk of bias [10–12, 21, 24], 3 studies had some concerns of bias [13, 23, 25], and 1 study had a high risk of bias [22] (Fig. 2).

### Adherence

Regarding supplement adherence, all but 2 studies [12, 22] provided details on how this was monitored and included: collection of used supplement containers [11, 13, 24, 25]; and adherence phone calls/interviews with research staff [10, 21, 23].

### Study summaries and outcomes

Fasting glucose was the most commonly measured of the specified outcomes (8 studies) [10–13, 21, 22, 24, 25], followed by HOMA-IR (8 studies) [10–13, 21, 23–25], fasting insulin (6 studies) [10–12, 21, 24, 25], and HbA1c (1 study) [13]. Detailed results from all studies for all reported primary outcomes are presented in Table 3.

**Table 3:** Outcome result details of the 9 included studies

Author (Year)	Intervention group	*n*	Primary outcome details (pre to post values ± SD)	Significant findings Intervention *v*s Control
Abootorabi et al. (2018) [10]	Intervention	22	HbA1c: NA HOMA-IR: 2.78 ± 1.31 to 2.82 ± 1.43 FPG: 86.74 ± 6.76 to 79.07 ± 7.09 FSI: 14.65 ± 7.45 to 15.92 ± 7.27	FPG ↓ No statistically significant difference in FSI or HOMA-IR
	Control	22	HbA1c: NA HOMA-IR: 1.69 ± 1.19 to 2.01 ± 0.67 FPG: 84.18 ± 5.85 to 85.90 ± 7.92 FSI: 8.19 ± 5.76 to 9.60 ± 3.30	
Ardabili et al. (2012) [21]	Intervention	30	HbA1c: NA HOMA-IR: 3.17 ± 4.08 to 3.21 ± 2.59 FSG: 99.79 ± 10.14 to 96.63 ± 9.87 FSI: 12.51 ± 15.13 to 13.34 ± 9.66	No statistically significant difference in FPG, FSI or HOMA-IR
	Control	30	HbA1c: NA HOMA-IR: 2.51 ± 1.41 to 2.46 ± 1.14 FSG: 101.50 ± 10.55 to 98.77 ± 14.62 FSI: 9.88 ± 5.26 to 9.98 ± 4.09	
Dastorani et al. (2018) [11]	Intervention	20	HbA1c: NA HOMA-IR: 2.5 ± 0.7 to 2.2 ± 0.7 FPG: 90.3 ± 10.5 to 89.4 ± 10.6 FSI: 11.2 ± 2.2 to 9.8 ± 2.7	FSI and HOMA-IR ↓ No statistically significant difference in FPG
	Control	20	HbA1c: NA HOMA-IR: 2.6 ± 0.5 to 2.5 ± 0.4 FPG: 92.9 ± 5.5 to 93.5 ± 5.6 FSI: 11.4 ± 1.9 to 11.1 ± 2.0	
Gupta et al. (2017) [22]	Intervention	25	HbA1c: NA HOMA-IR: 2.38 ± 4.88 to 1.00 ± 0.58 FSG: 88.24 ± 9.25 to 82.36 ± 8.03 FSI: 10.34 ± 20.00 to 5.00 ± 3.25	FSG, FSI and HOMA-IR ↓
	Control	25	HbA1c: NA HOMA-IR: NA FSG: NA FSI: NA	
Irani et al. (2015) [23]	Intervention	35	HbA1c: NA HOMA-IR: 2.07 ± 0.37 to 2.03 ± 0.22 FSG: NA FSI: NA	No statistically significant difference in HOMA-IR
	Control	18	HbA1c: NA HOMA-IR: 1.58 ± 0.30 to 1.52 ± 0.24 FSG: NA FSI: NA	
Javed et al. (2019) [24]	Intervention	18	HbA1c: NA HOMA-IR: 2.9 (2.8) to 2.5 (3.9) FPG: 84.6 (9.0) to 82.8 (12.6) FPI: 14.2 (12.8) to 12.3 (17.1)	No statistically significant difference in FPG, FPI or HOMA-IR
	Control	19	HbA1c: NA HOMA-IR: 2.1 (2.1) to 2.2 (2.8) FPG: 86.4 ± 7.2 to 86.4 ± 9.0 FPI: 11.7 ± 6.5 to 12.8 ± 8.0	
Maktabi et al. (2017) [25]	Intervention	35	HbA1c: NA HOMA-IR: 2.2 ± 1.1 to 1.8 ± 0.6 FPG: 91.0 ± 6.1 to 87.8 ± 7.6 FPI: 9.6 ± 4.5 to 8.2 ± 2.8	FPG, FPI and HOMA-IR ↓
	Control	35	HbA1c: NA HOMA-IR: 2.1 ± 1.7 to 2.7 ± 1.6 FPG: 93.8 ± 7.8 to 94.3 ± 9.8 FPI: 9.1 ± 7.3 to 11.7 ± 6.5	
Raja-Khan et al. (2014) [12]	Intervention	13	HbA1c: NA HOMA-IR: 5.47 ± 1.82 to 7.79 ± 7.37 FSG: 84.92 ± 9.46 to 83.82 ± 8.02 FSI: 26.31 ± 9.60 to 38.09 ± 37.60	No statistically significant difference in FPG, FPI or HOMA-IR
	Control	15	HbA1c: NA HOMA-IR: 5.80 ± 3.90 to 5.69 ± 2.97 FSG: 83.73 ± 9.33 to 77.64 ± 14.66 FSI: 27.13 ± 15.79 to 28.73 ± 14.64	
Trummer et al. (2019) [13]	Intervention	119	HbA1c: 33 (31–35) to 33 (32–35) HOMA-IR: 1.9 (1.1–3.5) to 2.3 (1.4–3.5) FPG: 84 ± 8 to 82 ± 8 FPI: NA	No difference in FPG, HbA1c or HOMA-IR
	Control	61	HbA1c: 34 (32–35) to 33 (32–35) HOMA-IR: 2.2 (1.3–3.0) to 2.3 (1.3–3.8) FPG: 84 ± 8 to 83 ± 7 FPI: NA	

Data are presented as mean ± SD if normally distributed, or median (interquartile range) if not normally distributed

*FPG* fasting plasma glucose (mg/dL); *FPI* fasting plasma insulin (μIU/mL); *FSG* fasting serum glucose (mg/dL); *FSI* fasting serum insulin (μIU/mL); *HbA1c* glycated haemoglobin; *HOMA-IR* homeostatic model assessment for insulin resistance; *IU* international units; *NA* not available

Abootorabi et al recruited 44 vitamin D deficient, Iranian women with PCOS for a randomized, single-blind, placebo-controlled trial. Participants received either vitamin D (50,000 IU/week) or placebo for 8 weeks [10]. Fasting glucose was reduced in the supplementation group (4.81 ± 0.38 to 4.39 ± 0.39 mmol/L, P = 0.001). However, there was no statistically significant change in fasting insulin or HOMA-IR.

60 vitamin D deficient women with PCOS were recruited by Ardabili et al for a randomized, double-blind, placebo-controlled trial conducted in Iran. Participants received either vitamin D (50,000 IU every 20 days) or placebo for 8 weeks [21]. The study found no statistically significant effect of vitamin D supplementation on measures of fasting glucose, fasting insulin or HOMA-IR.

Dastorani et al conducted a randomized, double-blind, placebo-controlled trial in Iran with 40 candidates for in vitro fertilization with PCOS [11]. Participants received either vitamin D (50,000 IU every 2 weeks) or placebo for 8 weeks. Vitamin D supplementation statistically significantly reduced fasting insulin ( – 1.4 ± 1.6 μIU/mL, P = 0.007) and HOMA-IR ( – 0.3 ± 0.3, P = 0.008) but had no statistically significant effect on fasting glucose.

The study by Gupta et al was a randomized, double-blind, placebo-controlled trial conducted in India with 50 women with PCOS [22]. Participants received either vitamin D (12,000 IU/week) or placebo for 12 weeks. Vitamin D supplementation statistically significantly reduced serum fasting glucose (88.24 ± 9.25 to 82.36 ± 8.03 mg/dl, P = 0.041), fasting insulin (10.34 ± 20.00 to 5.00 ± 3.25 μIU/mL, P = 0.021), and HOMA-IR (2.38 ± 4.88 to1.00 ± 0.58, P = 0.003).

In a randomized, single-blind, placebo-controlled trial conducted in the USA, Irani et al recruited 53 women with PCOS [23]. Participants received either vitamin D (50,000 IU/week) or placebo for 8 weeks. Vitamin D supplementation showed no statistically significant effect of HOMA-IR.

In a UK-based PCOS cohort, Javed et al recurited 37 women for a randomized controlled trial [24]. Participants received either vitamin D (3200 IU/day) or placebo for 12 weeks. Vitamin D supplementation did not significantly affect fasting glucose, fasting insulin, or HOMA-IR.

Maktabi et al conducted a randomized, double-blind, placebo-controlled trial in Iran with 70 women with PCOS [25]. Participants received either vitamin D (50,000 IU every 2 weeks) or placebo for 12 weeks. Vitamin D supplementation statistically significantly improved fasting glucose (5.05 ± 0.34 to 4.87 ± 0.42 mmol/L, P = 0.02), fasting insulin (reduced by 1.4 ± 3.6 μIU/ml, P = 0.004), and HOMA-IR (reduced by – 0.3 ± 0.8, P = 0.003).

In the USA, Raja-Khan et al conducted a randomized, double-blind, placebo-controlled trial with 30 women with PCOS [12]. Participants received either vitamin D (12,000 IU/day) or placebo for 12 weeks. High-dose vitamin D supplementation had no statistically significant effect on fasting glucose, fasting insulin or HOMA-IR.

In a randomized, double-blind, placebo-controlled trial conducted in Austria, Trummer et al recruited 180 women with PCOS [13]. Participants received either vitamin D (20,000 IU/week) or placebo for 24 weeks. Vitamin D supplementation did not statistically significantly affect fasting glucose, HOMA-IR, or HbA1c.

## Discussion

In the present study, I systematically reviewed RCTs investigating the effect of high-dose vitamin D supplementation on measures of glycemic control and insulin resistance, in women with PCOS. Analysis of all applicable studies revealed inconsistent results in terms of the effects of high-dose vitamin D supplementation on multiple measures of insulin resistance or glycemic control. Specifically, 5 out of the 9 studies identified did not observe any statistically significant improvements in either fasting glucose, fasting insulin, HOMA-IR or HbA1c [12, 13, 21, 23, 24]. Fasting glucose was observed to be reduced in the vitamin D supplementation group in 3 studies [10, 22, 25], fasting insulin was lowered in 3 studies [11, 22, 25] and HOMA-IR was lowered in 3 studies [11, 22, 25]. HbA1c was not statistically significantly reduced in any study.

The results of this review are in agreement with the results of a number of similar reviews. He et al performed a systematic review and meta-analysis with the aim of assessing both the association of serum vitamin D levels with metabolic dysregulations in women with PCOS, and to determine the effects of vitamin D supplementation on metabolic and hormonal functions in this population [27]. This review included all the measures of IR used in the present study as well as including homeostatic model assessment of β-cell function (HOMA-β) and quantitative insulin sensitivity check index (QUICKI), and found no evidence that vitamin D supplementation mitigated measures of insulin resistance in PCOS. It should be noted that this review did not specify criteria for dosages of vitamin D supplementation used in the included studies.

Some statistically significant effects of vitamin D supplementation have also been reported in systematic reviews. Łagowska et al performed a systematic review and meta-analysis comparing the effects of vitamin D supplementation alone or with co-supplements, with placebo, in women with PCOS [28]. Similarly to the present manuscript, the dosages in studies exclusively using vitamin D, included in this meta-analysis, ranged from 1000 IU/day to 60,000 IU/week. Co-supplementation interventions used lower doses. Vitamin D, when used alone, in doses below 4000 IU/d was seen to result in statistically significant reductions in HOMA-IR. This effect was not seen in interventions using more than 4000 IU/day and the authors speculated that this might be the result of the more regular absorption of vitamin D3 in the gut or better compliance with smaller, more regular doses. Statistically significant decreases in fasting glucose concentrations and HOMA-IR were reported only for interventions using Vitamin D when co-supplemented with other vitamins or minerals and not in interventions using vitamin D alone.

It is difficult to determine why some of the studies included in this systematic review show promising results while others do not, as this can likely be attributed to several factors. Differences in study design, sample size, duration, dosage, and baseline characteristics of participants may play a critical role in the variability of outcomes. For instance, varying individual doses of vitamin D ranged from 3200 to 50,000 IU with frequency of dosage ranging from once per day to once every 2 weeks. Additionally, the baseline vitamin D levels and insulin resistance status of participants, as well as study duration, may influence the effectiveness of the intervention. Furthermore, the geographical location and ethnic background of the study populations, which affect vitamin D metabolism and baseline deficiency levels, could also contribute to the observed discrepancies and may affect the reliability, validity and translatability of the findings. Furthermore, the existence of vitamin D receptor (VDR) gene polymorphisms may also be responsible for a variable response within individuals to vitamin D supplementation which may subsequently impact upon experimental outcomes. Studies have suggested that the TaqI VDR polymorphism and the FF genotype of the FokI variant may be associated with a better response to vitamin D supplementation [29]. Hence, it is crucial to consider these factors when interpreting the results of studies on vitamin D supplementation in women with PCOS.

Interest in the use of vitamin D as a possible treatment for IR in PCOS derives from research highlighting up to 70% of women with PCOS are vitamin D insufficient [9] and a similar proportion of women with PCOS exhibit some level of IR (Dunaif, 1997). The biological mechanisms by which vitamin D may influence insulin sensitivity are not entirely clear, although a number of potential mechanisms have been proposed. One proposed mechanism is that vitamin D increases calcium influx into pancreatic β-cells, enhancing insulin production [30]. As the interaction of vitamin D with the nuclear vitamin D receptor increases the efficiency of intestinal calcium absorption [31], impaired vitamin D status may lead to insufficient calcium status and subsequent impairment of β-cell insulin production. Indeed, previous research using 18 months of vitamin D supplementation (2000 IU/day), has reported improvements HOMA-β secretion (a measure of insulin secretion from pancreatic β-cells) in individuals with T2DM [32].

Another proposed mechanism posits that vitamin D may regulate insulin sensitivity through modulation of some insulin signalling pathways, particularly in skeletal muscle and adipose tissue. For example, research in rodent models has reported upregulated expression of vitamin d receptor (VDR) and insulin receptor substrate 1 (IRS-1) in skeletal muscle [33], and glucose transporter type 4 (GLUT4) in skeletal muscle cells [34], in response to vitamin D supplementation. IRS-1 is involved in regulation of insulin sensitivity and glucose homeostasis by modulation of the magnitude and duration of the insulin signalling response [35] and GLUT-4 is a glucose transporter which is involved in both insulin-stimulated and insulin independent glucose uptake in muscle and adipose tissue [36]. Thus, while the results of the present systematic review may be inconclusive, there is evidence for putative mechanisms by which vitamin D may affect insulin sensitivity.

This review has a number of strengths and limitations. A strength of this review is the focus on high-dose interventions using a minimum of 1000 IU/day of vitamin D. In fact, the lowest dose used in the included interventions was 1714 IU/day. Previous research has reported that doses of 1000–2000 IU/day of vitamin D3 over approximately 8-weeks may be necessary to achieve substantial changes in serum 25(OH)D levels, from a deficient state [19]. Despite recommended intakes of vitamin D being considerably lower (400 IU/day in the UK) [37], this dose may not be sufficient to induce changes in serum vitamin D levels nor subsequent physiological changes such as insulin sensitivity. Therefore, the inclusion of only high-dose vitamin D interventions in this review makes it more likely that the lack of effects observed is not due to insufficient supplementation.

Another factor worth consideration in this review is the inclusion of a number of studies with particularly severe vitamin D deficiency (< 30 nmol/L) [10, 11, 21, 24]. It might be assumed that those populations showing the greatest deficiency in serum vitamin D status would have the most to benefit from supplementation and thus be the most likely to benefit in terms of biomarkers of insulin resistance. However, statistically significant improvements in glucose, insulin and HOMA-IR were not consistent amongst these studies, with one [24] showing no statistically significant improvement in any of the measured markers.

There are also some limitations to this systematic review. Firstly, due to the inclusion/exclusion criteria used, the number of studies included in this systematic review was limited to nine, thus limiting the generalizability of the review’s findings as a smaller number of included studies may not be representative of the broader population. Furthermore, a smaller number of included studies limits the diversity of the included populations, making it challenging to draw definitive conclusions about the effects of the intervention in diverse groups [38]. However, the reason for maintaining the strict inclusion/exclusion criteria was to limit the possible heterogeneity of the included studies, thus strengthening the overall conclusions of the review within the context of those specific criteria.

Additionally, almost half (n = 4) of the studies included in the present systematic review were conducted in Iran where women are more likely to cover the majority of their skin thus reducing sunlight-stimulated vitamin D production and increasing the risk of vitamin D deficiency [39]. Therefore, results from these studies may not be extrapolated to populations that may receive more sun exposure, such as in the US and Europe.

It should also be noted that 4 of the included studies (almost half of the total) were determined to have some concerns or a high risk of bias [13, 22, 23, 25]. However, excluding these studies and focusing only on studies with a low risk of bias results in a total of 2 studies reporting statistically significant effects [10, 11], and a total of 3 studies reporting no statistically significant effects [12, 21, 24], thus not majorly altering the overall findings of this review.

Furthermore, it is important to clarify that the studies included in this systematic review, largely comply with the guidelines for clinical studies of nutrient effects proposed by Heaney [40]. Briefly, these state that (1) basal nutrient status must be measured; (2) the intervention must change the nutrient status; (3) this change must be measured and reported. However, other guidelines are not necessarily adhered to by all of these studies, such as (4) the hypothesis must be that a change in nutrient status produces the sought-for effect; and (5) conutrient status must be optimized in order to ensure that the test nutrient is the only nutrition-related, limiting factor in the response. However, due to the stringency of the guidelines for systematic reviews, put forward in the same paper [40] these guidelines were not followed in this manuscript and this should be considered a limitation of the systematic review.

Finally, while this review has focused on vitamin D, it is relevant to consider several other lifestyle factors known to significantly influence insulin resistance (IR) in women with PCOS. Importantly, pharmacological interventions such as metformin, and supplementation with inositol, both insulin-sensitizing agents, have been shown to improve insulin sensitivity and reduce insulin resistance in PCOS [41, 42]. Nutritional approaches, particularly low-glycemic index diets and those rich in fibre, can enhance insulin sensitivity and assist in weight management, a critical component in managing PCOS-related insulin resistance [43]. Regular physical activity, especially resistance and aerobic exercise, has been demonstrated to improve insulin sensitivity by enhancing glucose uptake and utilization in skeletal muscle [44]. Furthermore, adequate sleep duration and quality are crucial as sleep disturbances and poor sleep quality have been linked to increased IR and metabolic disturbances in PCOS [45]. Collectively, these lifestyle factors may play a vital role in the management of IR in PCOS, regardless of the potential benefits of vitamin D supplementation.

## Conclusion

In conclusion, in RCTs of high-dose vitamin D supplementation in women with PCOS, the majority of studies do not report statistically significant improvements in fasting glucose, fasting insulin, HbA1c or HOMA-IR. However, as a minority of studies report some statistically significant results, further investigation may be warranted.

## Supplementary Information

Below is the link to the electronic supplementary material.

Supplementary file1 (PDF 793 kb)

## Data Availability

Data sharing is not applicable to this article as no datasets were generated or analysed during the current study.

## Abbreviations

FPG: Fasting plasma glucose
FSG: Fasting serum glucose
FPI: Fasting plasma insulin
FSI: Fasting serum insulin
IR: Insulin resistance
IS: Insulin sensitvity
HbA1c: Glycated haemoglobin
HOMA-β: Homeostatic model assessment of β-cell function
HOMA-IR: Homeostatic model assessment for insulin resistance
QUICKI: Quantitative insulin sensitivity check index
RCT: Randomised controlled trial
T2DM: Type 2 diabetes mellitus

## Declarations

### Standards of reporting

The systematic review protocol was performed in accordance with the Preferred Reporting Items for Systematic Reviews and Meta-Analyses (PRISMA) statement guidelines.

## References

[1] Fauser BC, Tarlatzis BC, Rebar RW, Legro RS, Balen AH, Lobo R, Carmina E, Chang J, Yildiz BO, Laven JS (2012) Consensus on women’s health aspects of polycystic ovary syndrome (PCOS): the Amsterdam ESHRE/ASRM-sponsored 3rd PCOS consensus workshop group. Fertil Steril 97(1):28-38.e25 22153789 10.1016/j.fertnstert.2011.09.024

[2] Joham AE, Teede HJ, Ranasinha S, Zoungas S, Boyle J (2015) Prevalence of infertility and use of fertility treatment in women with polycystic ovary syndrome: data from a large community-based cohort study. J Women’s Health 24(4):299–307 10.1089/jwh.2014.5000 25654626

[3] Glintborg D, Rubin KH, Nybo M, Abrahamsen B, Andersen M (2018) Cardiovascular disease in a nationwide population of Danish women with polycystic ovary syndrome. Cardiovasc Diabetol 17(1):37. 10.1186/s12933-018-0680-5 29519249 10.1186/s12933-018-0680-5 PMC5844097

[4] Nestler JE (2000) Insulin resistance and the polycystic ovary syndrome: recent advances. Curr Opin Endocrinol Diabetes Obes 7(6):345–349

[5] Wimalawansa SJ (2018) Associations of vitamin D with insulin resistance, obesity, type 2 diabetes, and metabolic syndrome. J Steroid Biochem Mol Biol 175:177–189. 10.1016/j.jsbmb.2016.09.017 27662816 10.1016/j.jsbmb.2016.09.017

[6] Cashman KD, Dowling KG, Škrabáková Z, Gonzalez-Gross M, Valtueña J, De Henauw S, Moreno L, Damsgaard CT, Michaelsen KF, Mølgaard C, Jorde R, Grimnes G, Moschonis G, Mavrogianni C, Manios Y, Thamm M, Mensink GB, Rabenberg M, Busch MA, Cox L, Meadows S, Goldberg G, Prentice A, Dekker JM, Nijpels G, Pilz S, Swart KM, van Schoor NM, Lips P, Eiriksdottir G, Gudnason V, Cotch MF, Koskinen S, Lamberg-Allardt C, Durazo-Arvizu RA, Sempos CT, Kiely M (2016) Vitamin D deficiency in Europe: pandemic? Am J Clin Nutr 103(4):1033–1044. 10.3945/ajcn.115.120873 26864360 10.3945/ajcn.115.120873 PMC5527850

[7] Cashman KD (2020) Vitamin D deficiency: defining, prevalence, causes, and strategies of addressing. Calcif Tissue Int 106(1):14–29. 10.1007/s00223-019-00559-4 31069443 10.1007/s00223-019-00559-4

[8] Del Valle HB, Yaktine AL, Taylor CL, Ross AC (2011) Dietary reference intakes for calcium and vitamin D. Institute of Medicine, Food and Nutrition Board, Committee to Review Dietary Reference Intakes for Vitamin D and Calcium

[9] Wehr E, Pilz S, Schweighofer N, Giuliani A, Kopera D, Pieber TR, Obermayer-Pietsch B (2009) Association of hypovitaminosis D with metabolic disturbances in polycystic ovary syndrome. Eur J Endocrinol 161(4):575–582. 10.1530/eje-09-0432 19628650 10.1530/EJE-09-0432

[10] Abootorabi MS, Ayremlou P, Behroozi-Lak T, Nourisaeidlou S (2018) The effect of vitamin D supplementation on insulin resistance, visceral fat and adiponectin in vitamin D deficient women with polycystic ovary syndrome: a randomized placebo-controlled trial. Gynecol Endocrinol 34(6):489–494. 10.1080/09513590.2017.1418311 29271278 10.1080/09513590.2017.1418311

[11] Dastorani M, Aghadavod E, Mirhosseini N, Foroozanfard F, Modarres SZ, Siavashani MA, Asemi Z (2018) The effects of vitamin D supplementation on metabolic profiles and gene expression of insulin and lipid metabolism in infertile polycystic ovary syndrome candidates for in vitro fertilization. Reprod Biol Endocrinol. 10.1186/s12958-018-0413-3 30286768 10.1186/s12958-018-0413-3 PMC6172745

[12] Raja-Khan N, Shah J, Stetter CM, Lott MEJ, Kunselman AR, Dodson WC, Legro RS (2014) High-dose vitamin D supplementation and measures of insulin sensitivity in polycystic ovary syndrome: a randomized, controlled pilot trial. Fertil Steril 101(6):1740–1746. 10.1016/j.fertnstert.2014.02.021 24636395 10.1016/j.fertnstert.2014.02.021 PMC4537163

[13] Trummer C, Schwetz V, Kollmann M, Wölfler M, Münzker J, Pieber TR, Pilz S, Heijboer AC, Obermayer-Pietsch B, Lerchbaum E (2019) Effects of vitamin D supplementation on metabolic and endocrine parameters in PCOS: a randomized-controlled trial. Eur J Nutr 58(5):2019–2028. 10.1007/s00394-018-1760-8 29946756 10.1007/s00394-018-1760-8 PMC6647224

[14] Liberati A, Altman DG, Tetzlaff J, Mulrow C, Gotzsche PC, Ioannidis JP, Clarke M, Devereaux PJ, Kleijnen J, Moher D (2009) The PRISMA statement for reporting systematic reviews and meta-analyses of studies that evaluate health care interventions: explanation and elaboration. J Clin Epidemiol 62(10):e1-34. 10.1016/j.jclinepi.2009.06.006 19631507 10.1016/j.jclinepi.2009.06.006

[15] Higgins JPT, Thomas J, Chandler J, Cumpston M, Li T, P MJ, W VA (2019) Analysing data and undertaking meta-analyses. Cochrane Handb Syst Rev Interv. 10.1002/9781119536604.ch10

[16] Bonora E, Targher G, Alberiche M, Bonadonna RC, Saggiani F, Zenere MB, Monauni T, Muggeo M (2000) Homeostasis model assessment closely mirrors the glucose clamp technique in the assessment of insulin sensitivity: studies in subjects with various degrees of glucose tolerance and insulin sensitivity. Diabet Care 23(1):57–63. 10.2337/diacare.23.1.57 10.2337/diacare.23.1.57 10857969

[17] Abdul-Ghani MA, Matsuda M, DeFronzo RA (2008) Strong association between insulin resistance in liver and skeletal muscle in non-diabetic subjects. Diabet Med 25(11):1289–1294. 10.1111/j.1464-5491.2008.02597.x 19046218 10.1111/j.1464-5491.2008.02597.x

[18] Borai A, Livingstone C, Abdelaal F, Bawazeer A, Keti V, Ferns G (2011) The relationship between glycosylated haemoglobin (HbA1c) and measures of insulin resistance across a range of glucose tolerance. Scand J Clin and Lab Investig 71(2):168–172. 10.3109/00365513.2010.547947 21348785 10.3109/00365513.2010.547947

[19] Ginde AA, Liu MC, Camargo CA Jr (2009) Demographic differences and trends of vitamin D insufficiency in the US population, 1988–2004. Arch Intern Med 169(6):626–632. 10.1001/archinternmed.2008.604 19307527 10.1001/archinternmed.2008.604 PMC3447083

[20] Sterne JAC, Savović J, Page MJ, Elbers RG, Blencowe NS, Boutron I, Cates CJ, Cheng HY, Corbett MS, Eldridge SM, Emberson JR, Hernán MA, Hopewell S, Hróbjartsson A, Junqueira DR, Jüni P, Kirkham JJ, Lasserson T, Li T, McAleenan A, Reeves BC, Shepperd S, Shrier I, Stewart LA, Tilling K, White IR, Whiting PF, Higgins JPT (2019) RoB 2: a revised tool for assessing risk of bias in randomised trials. BMJ 366:l4898. 10.1136/bmj.l4898 31462531 10.1136/bmj.l4898

[21] Ardabili HR, Gargari BP, Farzadi L (2012) Vitamin D supplementation has no effect on insulin resistance assessment in women with polycystic ovary syndrome and vitamin D deficiency. Nutr Res 32(3):195–201. 10.1016/j.nutres.2012.02.001 22464806 10.1016/j.nutres.2012.02.001

[22] Gupta T, Rawat M, Gupta N, Arora S (2017) Study of effect of vitamin D supplementation on the clinical, hormonal and metabolic profile of the PCOS women. J Obstet Gynecol India 67:349–355 28867886 10.1007/s13224-017-1008-1 PMC5561752

[23] Irani M, Seifer DB, Grazi RV, Julka N, Bhatt D, Kalgi B, Irani S, Tal O, Lambert-Messerlian G, Tal R (2015) Vitamin D supplementation decreases TGF-beta 1 bioavailability in PCOS: a randomized placebo-controlled trial. J Clin Endocrinol Metab 100(11):4307–4314. 10.1210/jc.2015-2580 26485217 10.1210/jc.2015-2580

[24] Javed Z, Papageorgiou M, Deshmukh H, Kilpatrick ES, Mann V, Corless L, Abouda G, Rigby AS, Atkin SL, Sathyapalan T (2019) A randomized, controlled trial of vitamin D supplementation on cardiovascular risk factors, hormones, and liver markers in women with polycystic ovary syndrome. Nutrients. 10.3390/nu11010188 30658483 10.3390/nu11010188 PMC6356309

[25] Maktabi M, Chamani M, Asemi Z (2017) The effects of vitamin D supplementation on metabolic status of patients with polycystic ovary syndrome: a randomized, double-blind, placebo-controlled trial. Horm Metab Res 49(7):493–498. 10.1055/s-0043-107242 28679140 10.1055/s-0043-107242

[26] Biernacka-Bartnik A, Kocełak P, Owczarek AJ, Choręza PS, Markuszewski L, Madej P, Puzianowska-Kuźnicka M, Chudek J, Olszanecka-Glinianowicz M (2023) The cut-off value for HOMA-IR discriminating the insulin resistance based on the SHBG level in women with polycystic ovary syndrome. Front Med. 10.3389/fmed.2023.1100547 10.3389/fmed.2023.1100547 PMC10037532 36968815

[27] He C, Lin Z, Robb SW, Ezeamama AE (2015) Serum vitamin D levels and polycystic ovary syndrome: a systematic review and meta-analysis. Nutrients 7(6):4555–4577 26061015 10.3390/nu7064555 PMC4488802

[28] Łagowska K, Bajerska J, Jamka M (2018) The role of vitamin D oral supplementation in insulin resistance in women with polycystic ovary syndrome: a systematic review and meta-analysis of randomized controlled trials. Nutrients 10(11):1637 30400199 10.3390/nu10111637 PMC6266903

[29] Usategui-Martín R, De Luis-Román DA, Fernández-Gómez JM, Ruiz-Mambrilla M, Pérez-Castrillón JL (2022) Vitamin D receptor (VDR) gene polymorphisms modify the response to vitamin D supplementation: a systematic review and meta-analysis. Nutrients. 10.3390/nu14020360 35057541 10.3390/nu14020360 PMC8780067

[30] Gilon P, Chae H-Y, Rutter GA, Ravier MA (2014) Calcium signaling in pancreatic β-cells in health and in Type 2 diabetes. Cell Calcium 56(5):340–361. 10.1016/j.ceca.2014.09.001 25239387 10.1016/j.ceca.2014.09.001

[31] Holick MF (2007) Vitamin D deficiency. New England J Med 357(3):266–281 17634462 10.1056/NEJMra070553

[32] Al-Daghri NM, Alkharfy KM, Al-Othman A, El-Kholie E, Moharram O, Alokail MS, Al-Saleh Y, Sabico S, Kumar S, Chrousos GP (2012) Vitamin D supplementation as an adjuvant therapy for patients with T2DM: an 18-month prospective interventional study. Cardiovasc Diabetol 11(1):85. 10.1186/1475-2840-11-85 22809461 10.1186/1475-2840-11-85 PMC3461474

[33] Alkharfy KM, Al-Daghri NM, Yakout SM, Hussain T, Mohammed AK, Krishnaswamy S (2013) Influence of vitamin D treatment on transcriptional regulation of insulin-sensitive genes. Metab Syndr Relat Disord 11(4):283–288. 10.1089/met.2012.0068 23621113 10.1089/met.2012.0068

[34] Manna P, Achari AE, Jain SK (2017) Vitamin D supplementation inhibits oxidative stress and upregulate SIRT1/AMPK/GLUT4 cascade in high glucose-treated 3T3L1 adipocytes and in adipose tissue of high fat diet-fed diabetic mice. Arch Biochem Biophys 615:22–34. 10.1016/j.abb.2017.01.002 28063949 10.1016/j.abb.2017.01.002

[35] Gual P, Le Marchand-Brustel Y, Tanti JF (2005) Positive and negative regulation of insulin signaling through IRS-1 phosphorylation. Biochimie 87(1):99–109. 10.1016/j.biochi.2004.10.019 15733744 10.1016/j.biochi.2004.10.019

[36] Leto D, Saltiel AR (2012) Regulation of glucose transport by insulin: traffic control of GLUT4. Nat Rev Mol Cell Biol 13(6):383–396. 10.1038/nrm3351 22617471 10.1038/nrm3351

[37] Department of Health (1998) Nutrition and bone health: with particular reference to calcium and vitamin D. Report of the subgroup on bone health, working group on the nutritional status of the population of the committee on medical aspects of the food nutrition policy. Rep Health Soc Subj (Lond), vol 49, 1999/02/05 edn., 9932291

[38] Møller AM, Myles PS (2016) What makes a good systematic review and meta-analysis? BJA: Br J Anaesth 117(4):428–430. 10.1093/bja/aew264 28077528 10.1093/bja/aew264

[39] Vatandost S, Jahani M, Afshari A, Amiri MR, Heidarimoghadam R, Mohammadi Y (2018) Prevalence of vitamin D deficiency in Iran: a systematic review and meta-analysis. Nutr Health 24(4):269–278. 10.1177/0260106018802968 30296903 10.1177/0260106018802968

[40] Heaney RP (2014) Guidelines for optimizing design and analysis of clinical studies of nutrient effects. Nutr Rev 72(1):48–54. 10.1111/nure.12090 24330136 10.1111/nure.12090

[41] Lord JM, Flight IHK, Norman RJ (2003) Metformin in polycystic ovary syndrome: systematic review and meta-analysis. BMJ 327(7421):951. 10.1136/bmj.327.7421.951 14576245 10.1136/bmj.327.7421.951 PMC259161

[42] Greff D, Juhász AE, Váncsa S, Váradi A, Sipos Z, Szinte J, Park S, Hegyi P, Nyirády P, Ács N, Várbíró S, Horváth EM (2023) Inositol is an effective and safe treatment in polycystic ovary syndrome: a systematic review and meta-analysis of randomized controlled trials. Reprod Biol Endocrinol 21(1):10. 10.1186/s12958-023-01055-z 36703143 10.1186/s12958-023-01055-z PMC9878965

[43] Moran LJ, Ko H, Misso M, Marsh K, Noakes M, Talbot M, Frearson M, Thondan M, Stepto N, Teede HJ (2013) Dietary composition in the treatment of polycystic ovary syndrome: a systematic review to inform evidence-based guidelines. J Acad Nutr Diet 113(4):520–545. 10.1016/j.jand.2012.11.018 23420000 10.1016/j.jand.2012.11.018

[44] Hutchison SK, Stepto NK, Harrison CL, Moran LJ, Strauss BJ, Teede HJ (2011) Effects of exercise on insulin resistance and body composition in overweight and obese women with and without polycystic ovary syndrome. J Clin Endocrinol Metab 96(1):E48–E56. 10.1210/jc.2010-0828 20926534 10.1210/jc.2010-0828

[45] Zhang J, Ye J, Tao X, Lu W, Chen X, Liu C (2022) Sleep disturbances, sleep quality, and cardiovascular risk factors in women with polycystic ovary syndrome: systematic review and meta-analysis. Front Endocrinol. 10.3389/fendo.2022.971604 10.3389/fendo.2022.971604 PMC9513052 36176474

